# Alteration of tumor associated neutrophils by PIK3CA expression in endometrial carcinoma from TCGA data

**DOI:** 10.1186/s13048-019-0557-6

**Published:** 2019-08-31

**Authors:** Yinglian Pan, Li Ping Jia, Yuzhu Liu, Yixu Han, Qingchun Deng

**Affiliations:** 1 Department of Medical Oncology, Affiliated Hospital of Hainan Medical University, Haikou, Hainan 570102 People’s Republic of China; 2 Department of Gynecology, The Second Affiliated Hospital of Hainan Medical University, Haikou, Hainan 570102 People’s Republic of China

**Keywords:** Uterine corpus endometrial carcinoma, PIK3CA, Tumor associated neutrophils

## Abstract

Uterine corpus endometrial carcinoma (UCEC) is one of the most common cancer in female worldwide. PIK3CA has been proven to be a strong prognostic biomarker in UCEC. Nevertheless, current studies have not investigated what effects PIK3CA had on tumor associated neutrophils (TANss). Kaplan-Meier methods were used to compute the survival time of TCGA UCEC patients. GO and KEGG enrichment analysis unveiled relevant pathways PIK3CA affected using DEGs between PIK3CA high expression group and PIK3CA low expression group in TCGA UCEC, as well as GSEA. immune infiltration status was calculated using TIMER. We found that PIK3CA influenced a number of pathways including immune related pathways. The fraction of TANs was certainly altered by PIK3CA expression in UCEC. Our findings suggest that PIK3CA expression may play an important role in tumor immune microenvironment and could alter fraction of TANs in UCEC.

**Electronic supplementary material:**

The online version of this article (10.1186/s13048-019-0557-6) contains supplementary material, which is available to authorized users.

## Introduction

Uterine corpus endometrial carcinoma (UCEC) is the most common gynecologic malignancy in plenty of countries, 9 out of 10 women with early-stage disease present with the symptom of postmenopausal bleeding [1]. Over 50,000 women die from UCEC every year in the world [2]. UCSC disrupts a lot of signal pathways including PI3K/PTEN/AKT/mTOR pathway [3]. PI3K pathway serves a pivotal function that may have potential for defining targeted therapy for the treatment of grade 3 UCEC [4]. The main component of PI3K pathway, PIK3CA, is a strong prognostic biomarker in UCEC and associates with disease-specific mortality [5]. PIK3CA missense mutation is associated with unfavorable outcome in endometrioid carcinoma [6, 7]. PIK3CA and PIK3R1 mutations were frequent and showed a strong tendency for mutual exclusivity in UCSC [8]. The previous work on molecular mechanism of UCEC has indicated that PIK3CA played a crucial role in development of tumor.

Tumor-associated neutrophils (TANs) are phenotypically distinct from circulating neutrophils in terms of their surface protein composition and cytokines/chemokine activity, modulate the tumor immune microenvironment [9]. TANs can perform pro-tumoral functions, strengthening tumor cell invasion and metastasis, angiogenesis, and extracellular matrix remodeling, while inhibiting the antitumoral immune surveillance [10]. TANs recruit macrophages and Treg cells to hepatocellular carcinoma to promote their growth, progression, and resistance to sorafenib [11], which implied the regulated role of TANs in tumor immune microenvironment. High levels of TANs have been associated with a poor prognosis in different malignances [12]. TANs are an important component of the immune cell infiltrate in colorectal cancer and assessment of TAN infiltration may help identify patients likely to benefit from 5-FU-based chemotherapy [13]. Furthermore, TANs can also inhibit metastatic seeding in the lung cancer through hydrogen peroxide generation [14]. In general, the significant function of TANs has been verified in various researches.

However, until now no field research on what effects and internal mechanism PIK3CA has on TANs in UCEC have been reported. On the basis of existing literature data, we carried out studies in an effort to clarify the crucial role of PIK3CA in UCEC immune microenvironment.

## Results

### Expression level of PIK3CA was correlated with survival time of UCEC patients

**Fig. 1:**
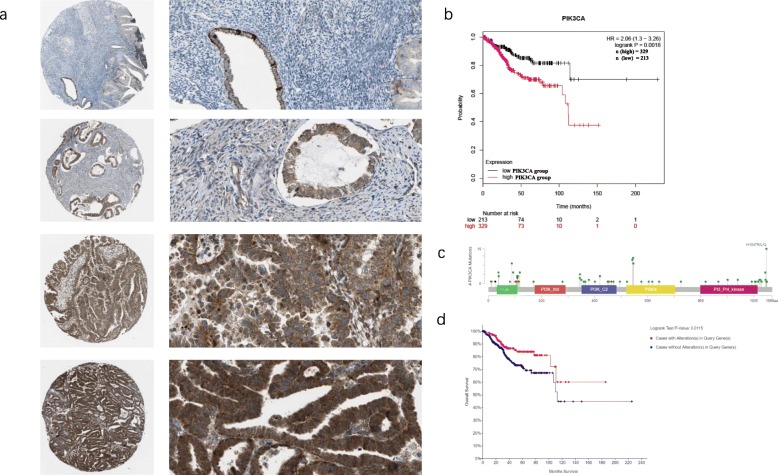
Expression levels of PIK3CA are correlated with survival of UCEC patients. **a** Immunohistochemical of PIK3CA in UCEC patients in Human Protein Atlas. The total number of UCEC patients screened for the analysis was 11. The histological subtypes of analyzed pathological section were endometrium adenocarcinoma. **b** Kaplan-Meier Survival Analysis of PIK3CA low expression group and PIK3CA high expression group. **c** Mutation sites and mutation types of PIK3CA gene among TCGA UCEC patients. **d** Survival analysis of patients with or without alteration in PIK3CA

The Human Protein Atlas showed the expression of PIK3CA in UCEC varied from medium to high and mainly in cytoplasm (Fig. 1a). Kaplan-Meier Survival Analysis [15] of 542 The Cancer Genome Atlas (TCGA) UCEC patients grouped by the expression of PIK3CA revealed a poorer clinical outcome in patients with high PIK3CA expression compared to those with low PIK3CA expression (*P* = 0.0018) (Fig. 1b). There were 170 mutation sites in TCGA UCEC patients PIK3CA gene analyzed by cBioPortal [16, 17], including 161 missense sites, 8 inframe sites, and 1 truncating sites (Fig. 1c). Patients without alteration in PIK3CA had poorer clinical outcome than those with alteration (*P* = 0.0115) (Fig. 1d). PIK3CA expression could influence survival time of UCEC patients, which suggested its prognosis.

### A lot of molecules were related to PIK3CA expression in UCEC

**Fig. 2:**
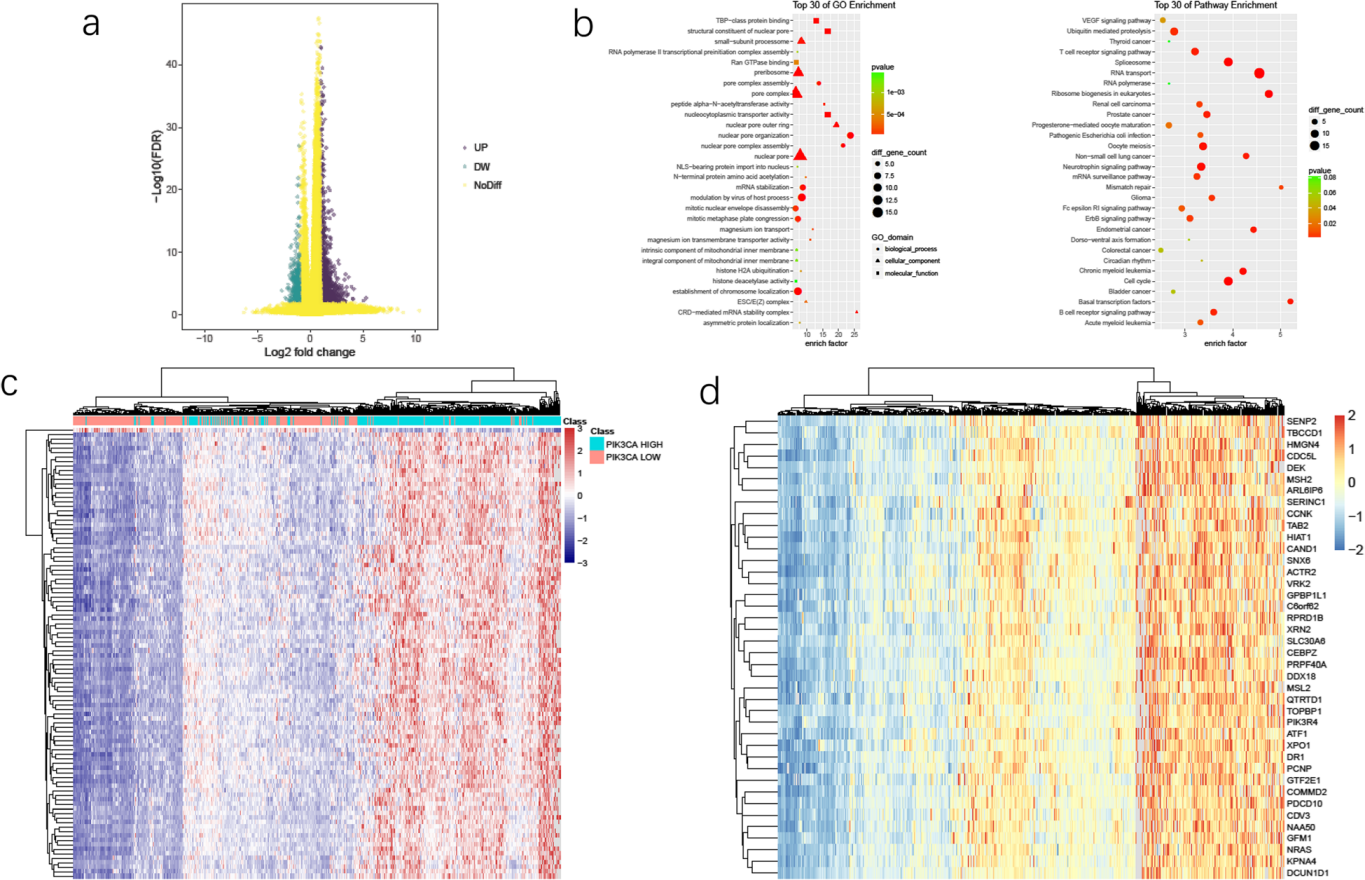
DEGs and enrichment analysis between PIK3CA low group and PIK3CA high group. **a** Volcano plot of DEGs between PIK3CA low group and PIK3CA high group. Purple dots represent up-regulated genes in PIK3CA high group (log_2_FC > 1, FDR < 0.01), whereas green dots represent up-regulated genes in PIK3CA low group (log_2_FC > 1, FDR < 0.01). **b** GO and KEGG enrichment analysis of top 500 DEGs. **c, d** Heatmap of DEGs between PIK3CA low group and PIK3CA high group

We divided TCGA UCEC patients into two subgroups by PIK3CA relative expression, PIK3CA low group (*n* = 255) and PIK3CA high group (*n* = 256). By setting log_2_(Fold change) as ±1 and FDR as 0.01, we identified 4315 Different Expressing Genes (DEGs) between PIK3CA low group and PIK3CA high group (Fig. 2a). There were 3623 upregulated DEGs and 692 downregulated DEGs.

There were a variety of genes that expressed dissimilarly between PIK3CA low group and PIK3CA high group, which implied these genes played significant roles in the development of UCEC through PIK3CA (Fig. 2c, d). In addition, a number of genes got clustered and collectively expressed higher in the majority of PIK3CA high group patients (Fig. 2c). This phenomenon showed that a set of genes may get together to influence PIK3CA expression.

### GO and KEGG enrichment analysis unveiled pathways PIK3CA affected in UCEC

In order to find pathways PIK3CA affects, we applied GO and KEGG enrichment analysis using top 500 DEGs (Fig. 2b).

GO enrichment analysis showed plenty of pathways were well enriched, including CRD-mediated mRNA stability complex, nuclear pore organization, nuclear pore complex assembly, nuclear pore outer ring, structural constituent of nuclear pore, nucleocytoplasmic transporter activity, peptide alpha-N-acetyltransferase activity, pore complex assembly, TBP-class protein binding, magnesium ion transport (Fig. 2b, left). Most of these pathways belonged to biological process and cellular component related pathways.

KEGG enrichment analysis showed some pathways were enriched significantly, including Basal transcription factors, Mismatch repair, Ribosome biogenesis in eukaryotes, RNA transport, Endometrial cancer, Non-small cell lung cancer, Chronic myeloid leukemia, Cell cycle, Spliceosome (Fig. 2b, right). PIK3CA expression could affect multiple tumor progression as reported [18–20]. Besides, B cell receptor signaling pathway (*P* = 0.002, enrich factor = 3.6), T cell receptor signaling pathway (*P* = 0.001, enrich factor = 3.21) and Natural killer cell mediated cytotoxicity (*P* = 0.06, enrich factor = 1.98) were well enriched, which suggested PIK3CA expression might have an effect on immune system.

In addition, top 800 DEGs KEGG enrichment analysis showed another immune related pathway, mTOR signaling pathway, was significant enriched (*P* = 0.013, enrich factor = 4.2) (Additional file 1: Figure S1).

### PIK3CA expression significantly influenced neutrophil related pathway

**Fig. 3:**
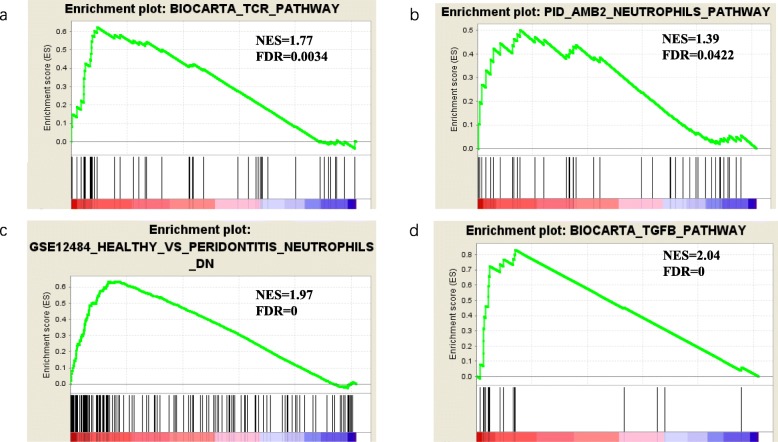
GSEA analyzing PIK3CA low group and PIK3CA high group expression data in four pathways including (**a**) BIOCARTA TCR PATHWAY, (**b**) PID AMB2 NEUTROPHILS PATHWAY, (**c**) GSE12484 HEALTHY VS PERIDONTITIS NEUTROPHILS DN and (**d**) BIOCARTA TGFB PATHWAY

Next, we utilize Gene Set Enrichment Analysis (GSEA) to enrich in immune related pathways using PIK3CA low group and PIK3CA high group expression data. The T Cell Receptor plays a key role in the immune system. As can be seen from Fig. 3a, PIK3CA high group was enriched in T Cell Receptor Signaling Pathway (NES = 1.77, FDR = 0.0034), which is consistent with the observation from KEGG enrichment analysis (Fig. 2b). It must also be mentioned that PIK3CA high group enriched significantly in two neutrophil related pathways (NES = 1.39 FDR = 0.0422, NES = 1.97 FDR = 0, respectively) (Fig. 3b, c). Identical result were obtained in TGFβ pathway (NES = 2.04, FDR = 0) (Fig. 3d), which plays an important role in tumor initiation and progression and increases neutrophil-attracting chemokines resulting in recruitment and activation of neutrophils with an antitumor phenotype [21]. These results provide substantial evidence for the above assumptions that PIK3CA expression might have an effect on immune system, more probably, on TANs.

By the way, two famous tumor related pathways were well enriched, Wnt Pathway (NES = 1.89, FDR = 0.0001) and JNK MAPK Pathway (NES = 2.1, FDR = 0) (Additional file 2; Figure S2a, S2b). MTOR Pathway (NES = 1.79, FDR = 0.0007) and CD40 Pathway (NES = 1.76, FDR = 0.0012) were also significantly enriched, which indicated a good agreement with our assumption.

### Immune infiltration analysis suggested TANs were influenced by PIK3CA expression

**Fig. 4:**
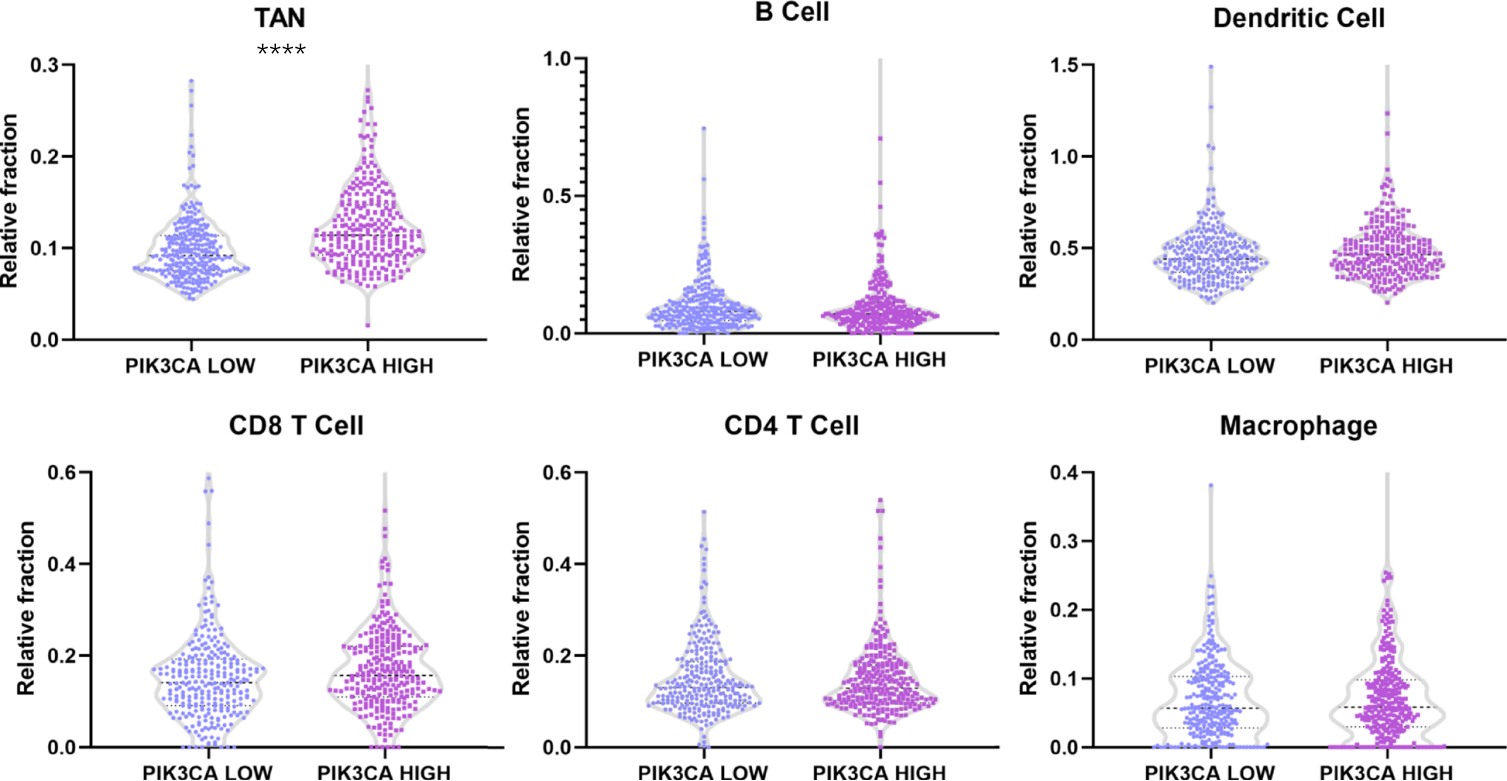
Violin plots of TANs, B cells, dendritic cells, CD4 T cells, CD8 T cells and macrophages relative fraction in PIK3CA low group and PIK3CA high group analyzing by online tools TIMER (****: *P* < 0.0001)

UCEC immune infiltration status was calculated using TIMER, A Web Server for Comprehensive Analysis of Tumor-Infiltrating Immune Cells. As shown in Fig. 4, there was a significant increase in TANs in PIK3CA high group compare with PIK3CA low group (*P* < 0.0001), which was in good consistent with the KEGG enrichment analysis and GSEA. On the contrary, relative fraction of B cells, dendritic cells, CD4 T cells, CD8 T cells and macrophages showed no difference between two groups (*P* > 0.05). Our findings suggest that PIK3CA may play a key role in alteration of TANs in UCEC. And high TANs predicted poor prognosis in UCEC according to our analysis using TCGA UCEC clinical follow-up information (*p* = 0.09) (Additional file 3: Figure S3).

### TANs related gene expression confirmed TANs modification by the influence of PIK3CA expression

**Fig. 5:**
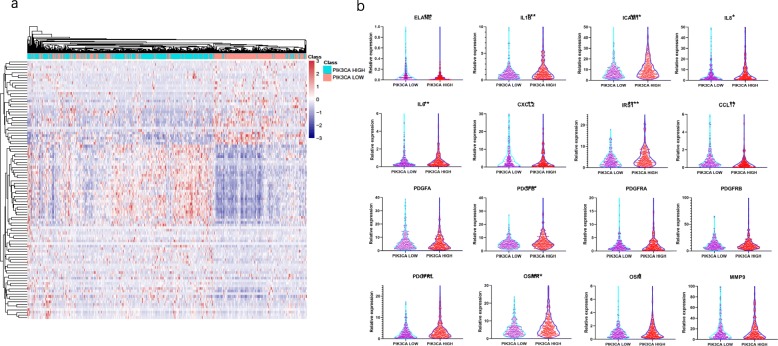
Expression of neutrophil related genes in PIK3CA low group and PIK3CA high group. **a** Heatmap of neutrophil related genes expression. **b** Violin plots of key neutrophil related genes expression (*: *P* < 0.05, **: *P* < 0.01, ***: *P* < 0.001, ****: *P* < 0.0001)

Finally, we verified the increase of TANs in PIK3CA high group by assessing TANs related gene expression. A cluster of TANs related genes expressed higher in PIK3CA high group, while the other cluster expressed higher in PIK3CA low group (Fig. 5a). Multiple TANs related gene expression formed a unique pattern in two groups, together to alteration the amount of TANs. TANs express several molecular markers [9], most of which express significantly differently between PIK3CA low group and PIK3CA high group, including ELANE (FDR = 0.0002), IL1B (FDR = 0.0009), ICAM1 (FDR < 0.0001), IL8 (FDR = 0.041), IL6 (FDR = 0.0027), CXCL2 (FDR = 0.0061), IRS1 (FDR < 0.0001), CCL17 (FDR = 0.0019), PDGFRA (FDR = 0.034), PDGFRL (FDR < 0.0001), OSMR (FDR < 0.0001), OSM (FDR = 0.011) (Fig. 5b).

## Discussion

UCEC is one of the most common cancer in female worldwide. Determining what factors cause UCEC progressing predominantly has been a subject of intense interest. Meanwhile, PIK3CA was proven to be a significant molecule during the progression of UCEC. Our result showed PIK3CA expressed differently, ranging from medium to high, in UCEC patients, which suggested tumor progression could be largely influenced PIK3CA expression quantity. PIK3CA, as expected, could change the outcome of UCEC patients, leading to shorter survival time in high PIK3CA group.

A lot of genes related to PIK3CA found in our research has been reported to correlate with other cancer types, which proved the importance of PIK3CA. PIK3R4 mutation was discovered in metastatic melanoma [22], suggested an important function in melanoma. Meanwhile, copy number gains of PIK3CA and PIK3R4 were associated with decreased survival in ovarian cancer [23]. PIK3R4 might alter PIK3CA expression in ovarian cancer. SENP2 have not been reported in UCEC related research. However, it may play a crucial role in hypoxia-induced lung cancer progression [24] and diagnosis or therapeutic targets for breast cancer treatment [25]. TBCCD1, a key regulator of centrosome positioning and consequently of internal cell organization, is a new centrosomal protein [26], and it has not been mentioned in any cancer.

Multiple kinds of cancer including thyroid cancer, prostate cancer, non-small cell lung cancer, glioma, colorectal cancer, chronic and acute myeloid leukemia, endometrial cancer and renal cell carcinoma seemed to affect by PIK3CA expression, some of which was demonstrated in a number of studies. PIK3CA may play a significant role in tumor cell proliferation, invasion, metastasis, apoptosis or cell cycle among different cancer types. It is worthwhile mentioning that PIK3CA probably regulates some common molecules or pathways in different cancer. Targeting PIK3CA to investigate and develop drugs may be a new way for better treatment of cancer, including UCEC.

Immunotherapy of cancer has become the center of attention these years. Most researches were related to T cell or B cell, whereas few reports were related to TANs. However, TANs played an important part in immune microenvironment of many cancer types. It should be pointed out that no one has investigated TANs in UCEC. Our findings suggest that fraction of TANs are significantly altered by PIK3CA expression in UCEC, which is crucial for further studies of immune microenvironment and immunotherapy in UCEC.

The novelty of the study is that it was the first time and research to find out intrinsic mechanism in which PIK3CA influence TANs in UCEC. Our result implied the potential role of PIK3CA or other components of PI3K pathway in tumor immune microenvironment and may open a new approach for clinical anti-tumor research and anti-tumor drug development.

The shortcoming of our research was that we have not tested results in vivo owing to limitation of experimental conditions. However, we proposed a new aspect in UCEC immune infiltration status, which was influenced by PIK3CA expression.

Further effort is required to confirm the specific mechanism PIK3CA engaged to alter the fraction of TANs in UCEC. There is thereby an urgent need but it is still a significant challenge to uncover the role in immune system in UCEC.

## Method

Acquisition and processing of TCGA UCEC patients’ mRNA-seq data.

mRNA-seq FPKM data of TCGA UCEC patients was downloaded from The Cancer Genome Atlas. All UCEC patients were divided into two subgroups according to the expression of PIK3CA. PIK3CA low group contained 255 patients, whereas PIK3CA high group contained 256 patients. DEGs were calculated by setting log_2_(Fold change) as ±1 and FDR as 0.01. Utilizing ImageGP (http://www.ehbio.com/ImageGP/) drew Volcano plot. Heatmap was generated by R (3.3.2) pheatmap package.

GO and KEGG enrichment analysis and GSEA.

Top 500 DEGs selected to process GO and KEGG enrichment analysis. GO and KEGG enrichment analysis were performed and visualized on website (http://enrich.shbio.com/index/ga.asp). Gene Set Enrichment Analysis (GSEA) is a computational method that determines whether an a priori defined set of genes shows statistically significant, concordant differences between two biological states. UCEC mRNA-seq data were divided into PIK3CA low group and PIK3CA high group As mentioned and performed GSEA. Several pathways were selected to analyze including Wnt Pathway, JNK MAPK Pathway, MTOR Pathway, CD40 Pathway using GSEA. NES and FDR were selected to value the pathway enrichment in each group. GSEA was performed using the GSEA 3.0 software.

### Immune infiltration analysis

Immune infiltration status was analyzed by TIMER (https://cistrome.shinyapps.io/timer/), visualized by GraphPad Prism 8. We download TCGA tumor immune estimation data from website and filtered the UCEC PIK3CA low group and PIK3CA high group data for immune infiltration analysis. Percents of B cells, CD4 T cells, CD8 T cells, TANs, macrophages and dendritic cells in PIK3CA low group and PIK3CA high group were compared using t test.

### Survival analysis

Survival analysis of 542 TCGA UCEC patients was performed on Kaplan-Meier Survival Analysis [15] (http://kmplot.com/analysis/). 542 patients were divided into two subgroups, PIK3CA low group (*n* = 213) and PIK3CA high group (*n* = 329). Survival of UCEC patients with high TANs and low TANs was illustrated with the help of TCGA UCEC clinical follow-up information.

### Immunohistochemical images acquisition

Images of PIK3CA antibody stained UCEC tissue were obtained on Human Protein Atlas (https://www.proteinatlas.org/). The total number of UCEC patients screened for the analysis was 11. The histological subtypes of analyzed pathological section were endometrium adenocarcinoma.

### cBioPortal

One hundred seventy mutation sites in TCGA UCEC patients PIK3CA gene were analyzed by cBioPortal (http://www.cbioportal.org/), so as to survival status.

## Additional files

Additional file 1: **Figure S1.** GO and KEGG enrichment analysis of top 800 DEGs. (PDF 86 kb)

Additional file 2: **Figure S2.** GSEA analyzing PIK3CA low group and PIK3CA high group expression data in four pathways. (PDF 68 kb)

Additional file 3: **Figure S3.** Survival of UCEC patients with high TANs and low TANs. (PDF 10 kb)

## Data Availability

All mRNA-seq FPKM data is available in The Cancer Genome Atlas.

## Abbreviations

DEGs: Different Expressing Genes
GSEA: Gene Set Enrichment Analysis
TANs: Tumor Associated Neutrophils
TCGA: The Cancer Genome Atlas
UCEC: Uterine Corpus Endometrial Carcinoma
