# Supplementary figures and images for: Alteration of tumor associated neutrophils by PIK3CA expression in endometrial carcinoma from TCGA data
Source: J Ovarian Res. 2019 Aug 31;12:81. doi: 10.1186/s13048-019-0557-6 (PMC6717327; doi:10.1186/s13048-019-0557-6)

a

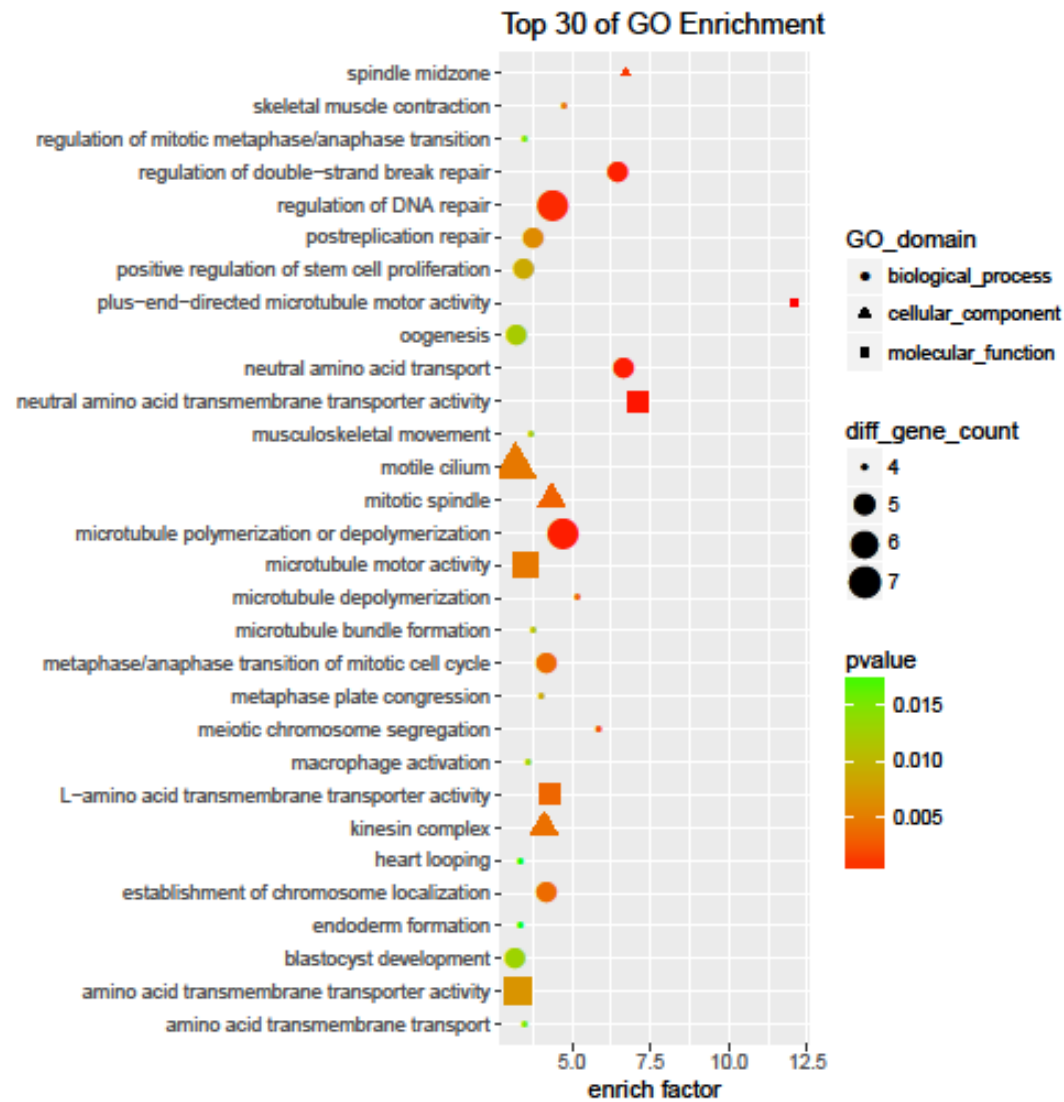

b

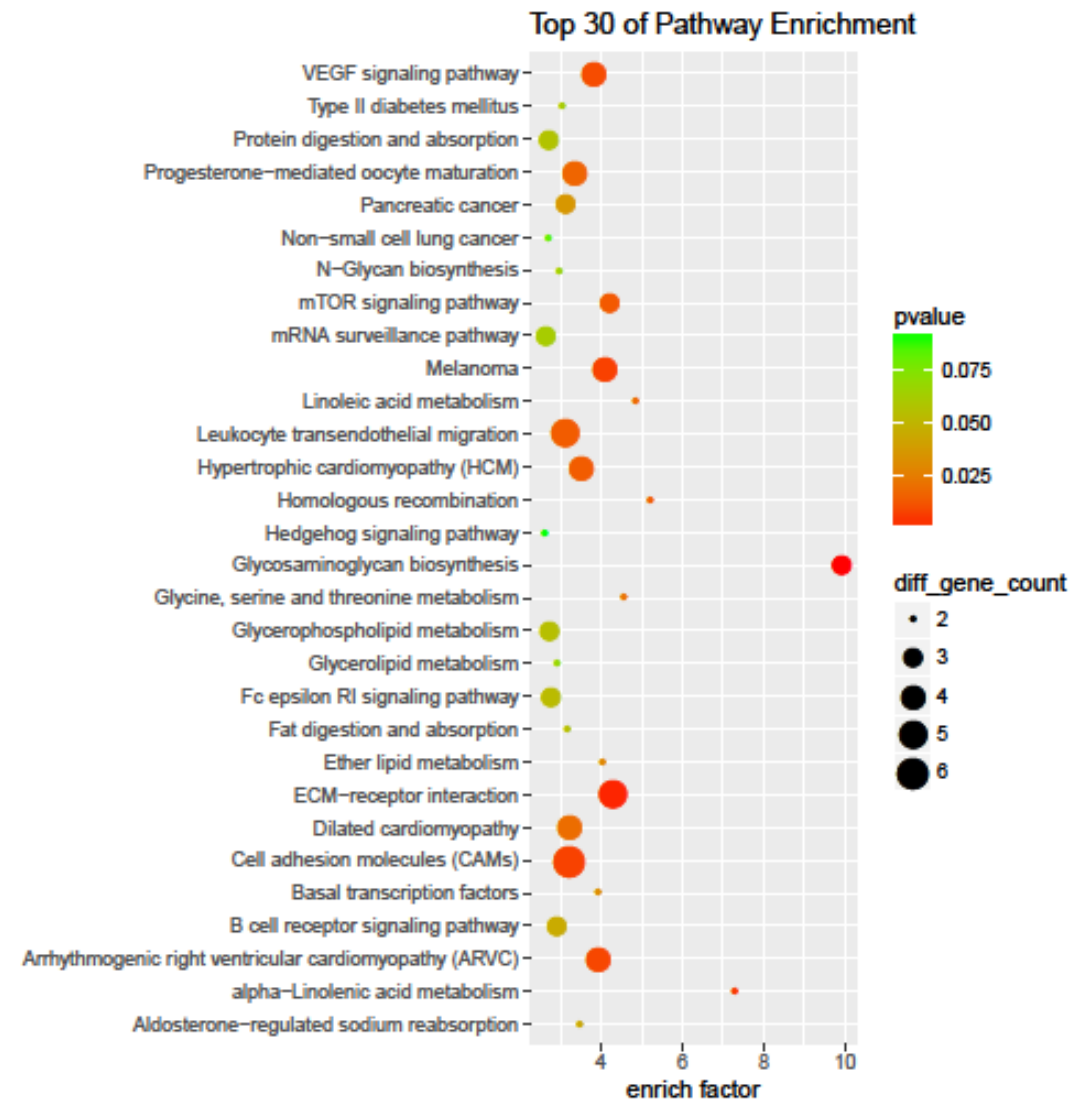

Supplement: Supplementary file 1 — Figure S1. GO and KEGG enrichment analysis of top 800 DEGs. (PDF 86 kb) [file 13048_2019_557_MOESM1_ESM.pdf]

a

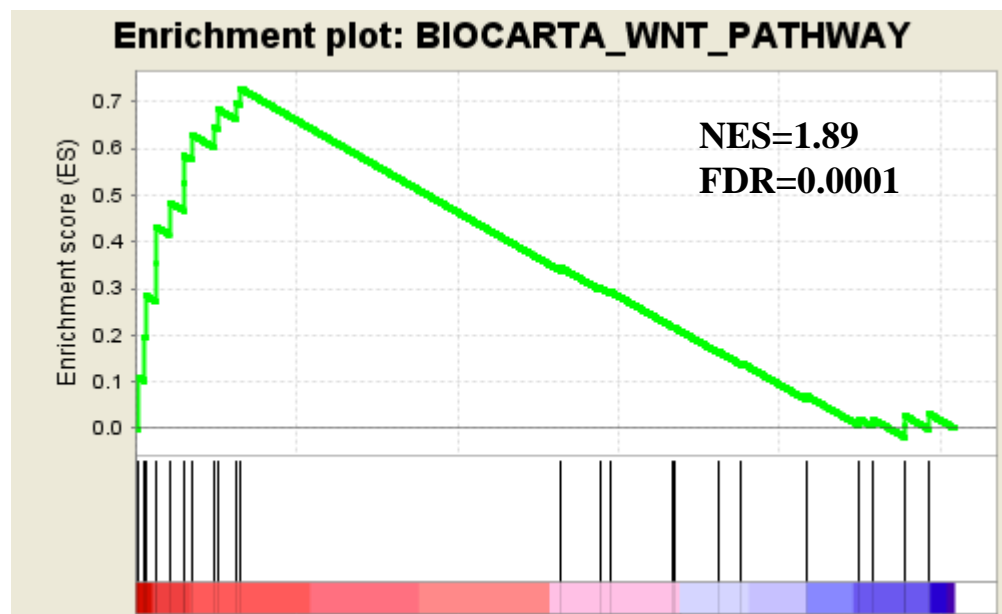

b

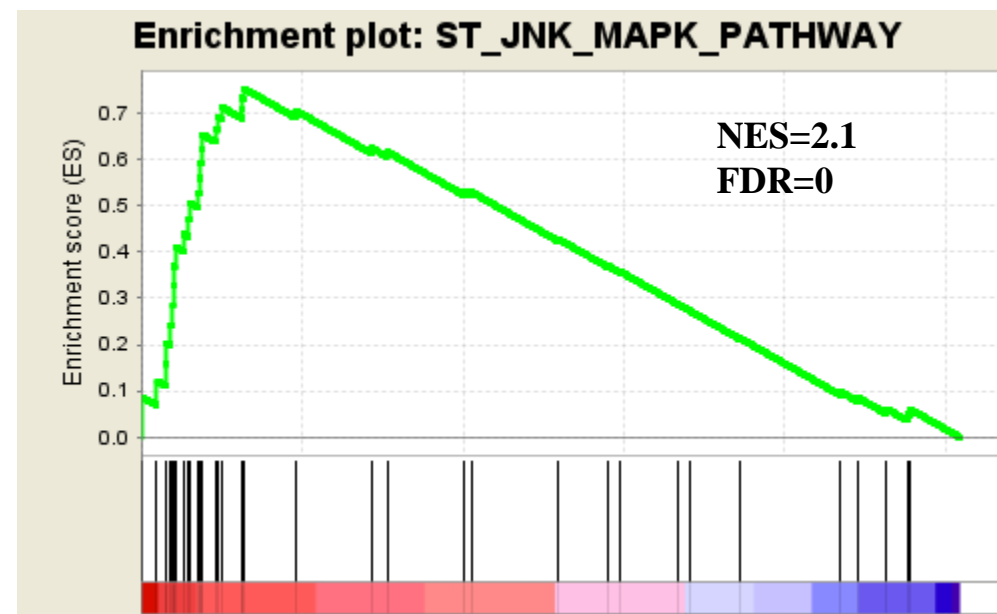

c

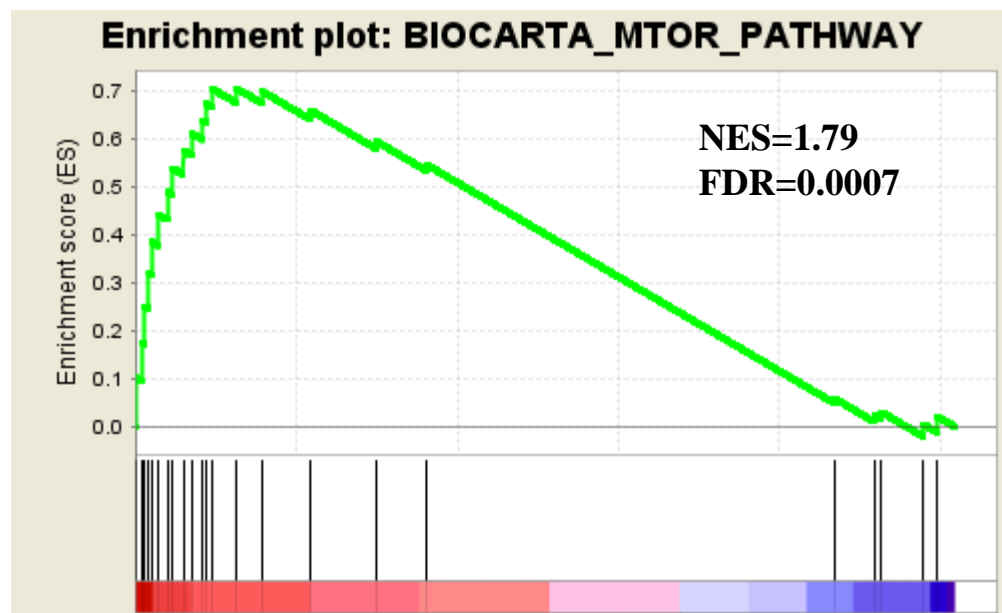

d

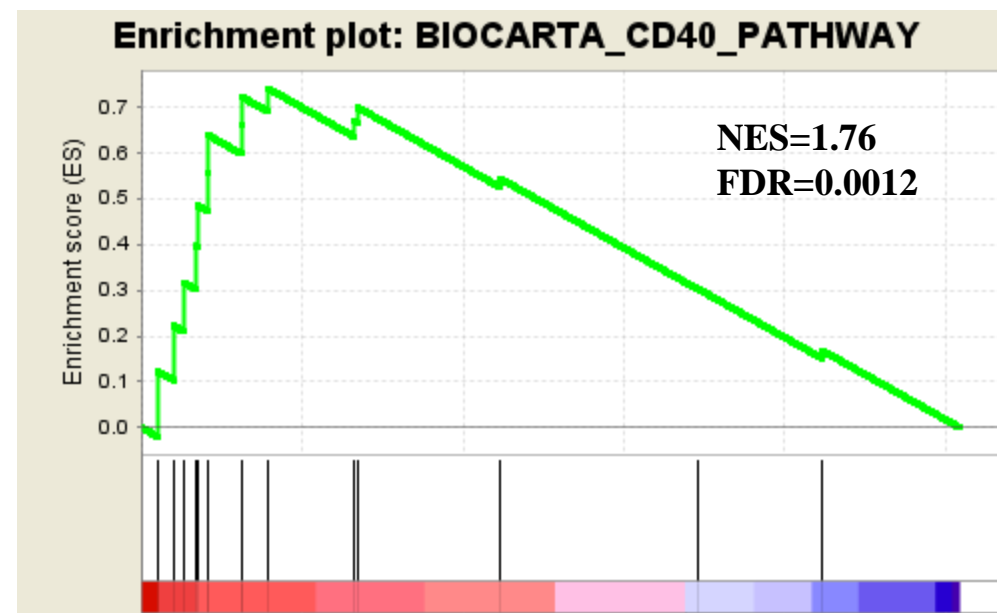

Supplement: Supplementary file 2 — Figure S2. GSEA analyzing PIK3CA low group and PIK3CA high group expression data in four pathways. (PDF 68 kb) [file 13048_2019_557_MOESM2_ESM.pdf]

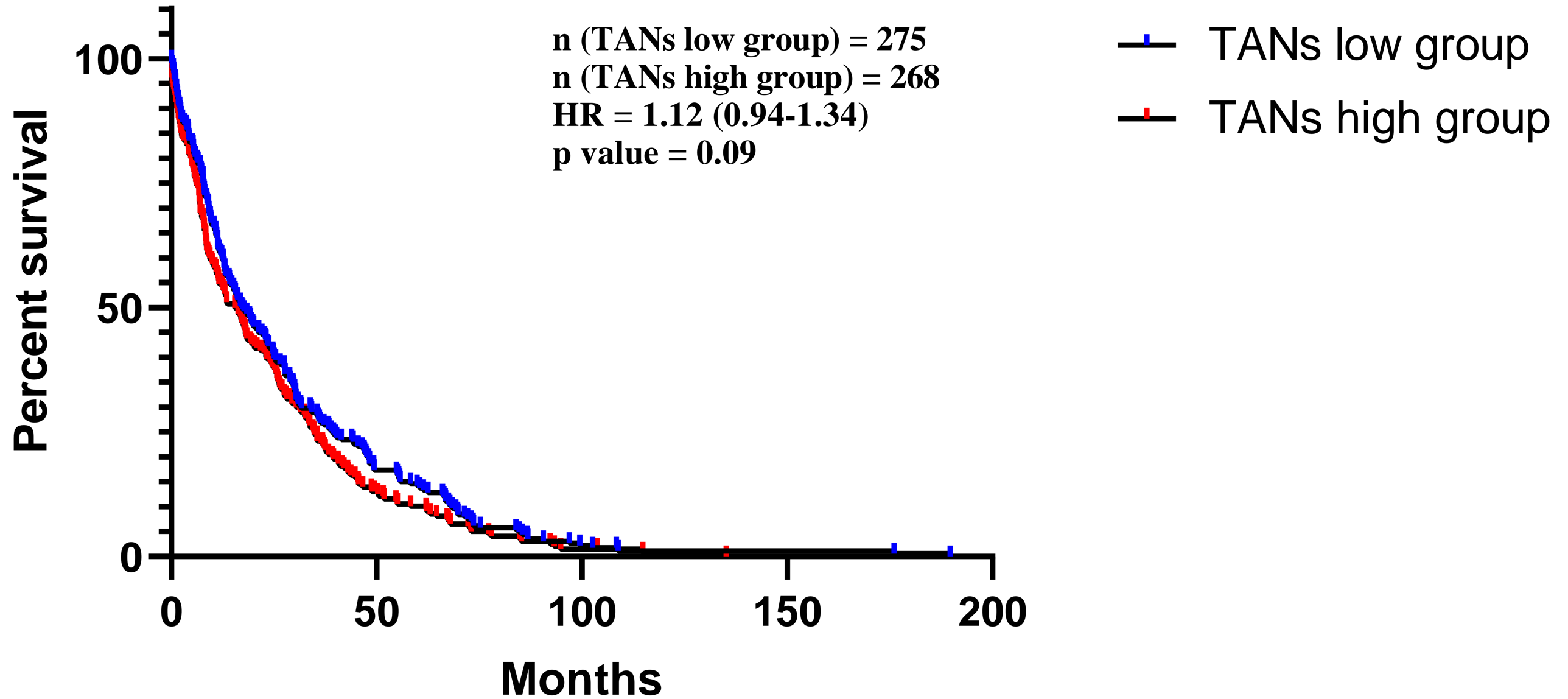

Supplement: Supplementary file 3 — Figure S3. Survival of UCEC patients with high TANs and low TANs. (PDF 10 kb) [file 13048_2019_557_MOESM3_ESM.pdf]
